# Sphingosine 1-Phosphate: A Novel Target for Lung Disorders

**DOI:** 10.3389/fimmu.2017.00296

**Published:** 2017-03-14

**Authors:** Sabira Mohammed, K. B. Harikumar

**Affiliations:** ^1^Cancer Research Program, Rajiv Gandhi Centre for Biotechnology, Thiruvananthapuram, India; ^2^Manipal Academy of Higher Education, Manipal, India

**Keywords:** sphingosine kinase, sphingosine 1-phosphate, S1PR, lung diseases, asthma, FTY720

## Abstract

Sphingosine 1-phosphate (S1P) is involved in a wide range of cellular processes, which include proliferation, apoptosis, lymphocyte egress, endothelial barrier function, angiogenesis, and inflammation. S1P is produced by two isoenzymes, namely, sphingosine kinase 1 and 2 (SphK1 and 2) and once produced, S1P can act both in an autocrine and paracrine manner. S1P can be dephosphorylated back to sphingosine by two phosphatases (SGPP 1 and 2) or can be irreversibly cleaved by S1P lyase. S1P has a diverse range of functions, which is mediated in a receptor dependent, through G-protein coupled receptors (S1PR1–5) or receptor independent manner, through intracellular targets such as HDACs and TRAF2. The involvement of S1P signaling has been confirmed in various disease conditions including lung diseases. The SphK inhibitors and S1PR modulators are currently under clinical trials for different pathophysiological conditions. There is a significant effort in targeting various components of S1P signaling for several diseases. This review focuses on the ways in which S1P signaling can be therapeutically targeted in lung disorders.

## Introduction

**Figure 1:**
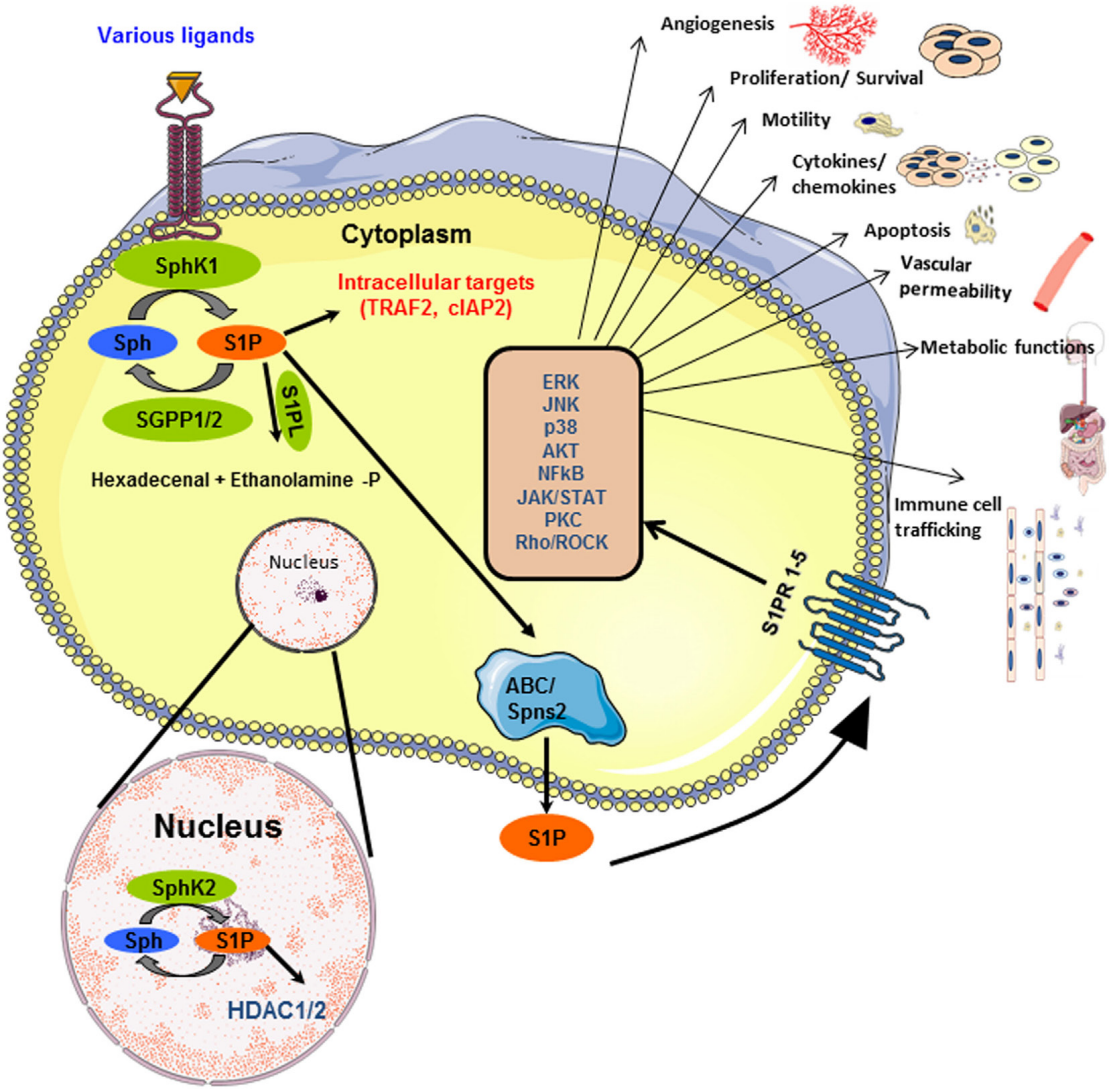
**Inside-out signaling of sphingosine 1-phosphate (S1P): SphK1 and SphK2 get activated by various ligands, in the cytoplasm and nucleus, respectively, and lead to the production of S1P**. The schematic representation of the different fates of S1P is shown. S1P can be dephosphorylated to sphingosine by phosphatases (SGPP1/2) or irreversibly cleaved into hexadecenal and ethanolamine phosphate by S1P lyase. S1P, transported outside the cell by ABC transporters or Spns2, binds to any of the five G-protein-coupled receptors (S1PR1–5) on cell membrane. This activates several downstream signaling pathways. Besides this, S1P interacts with proteins such as TRAF2 in the cytoplasm and HDAC1/2 in the nucleus, thereby functioning as an intracellular second messenger.

Sphingolipids are ubiquitous components of the cell membrane and provide structural integrity. Ceramide, one of the simplest structural sphingolipids, is converted to sphingosine which in turn is phosphorylated by two isoenzyme specific kinases, namely, SphK1 and 2 to form sphingosine 1-phosphate (S1P) (1–3). There is a tight regulation of formation and degradation of S1P within the cells, through activation of SphKs to form S1P, dephosphorylation by phosphatases (SPPs) or degradation by S1P lyase (SPL) (Figure 1). Research over the past two decades identified S1P as a potent bioactive lipid molecule that regulates various cellular processes including cell growth, apoptosis, immune regulation, etc. (4–9).

SphK/S1P axis has been implicated in several diseases and is often considered as a potential therapeutic target. The level of expression of SphK1, and thereby S1P, has been found to correlate with the disease grade, severity, and patient survival. The involvement of sphingolipids varies from cardiovascular, cancer, inflammatory diseases to obesity.

Lung diseases are broadly classified into (a) airway diseases, where the airways, which carry oxygen and other gases, are affected; (b) lung tissue diseases, which mainly affect the architecture of lung; and (c) lung circulation/pulmonary diseases, which mainly affect the blood vessels in lungs. The involvement of S1P signaling in different types of lung diseases is currently an active area of research. We summarized the effects mediated by S1P signaling in lung diseases in the following sections.

### Asthma

The recent statistics provided by Global Asthma Network indicated that the number of people diagnosed with Asthma is increasing worldwide (10). The first study about the role of S1P was reported by Choi’s group using rat mast-cell line (RBL-2H3) and showed that crosslinking and stimulation of FcεRI (high-affinity IgE receptor) could activate SphK1 and subsequently lead to S1P production (11). Conversely, high intracellular levels of sphingosine can block mast cell activation. Differential role of SphK1 and 2 has been observed in mast cells. Though both the isoforms are needed for production of TNF, SphK1 plays a key role in degranulation and migration towards antigen (12). S1P levels were increased in the airways of asthmatic patients but not in control subjects. Moreover, S1P also regulates the functions of airway smooth muscles during inflammation and airway remodeling (13). One of the mechanisms is through the inhibition of myosin phosphatase by S1P through a RhoA-dependent mechanism (14). Muscarinic receptor (MR) signaling that regulates airway resistance has been implicated in asthma (15). The MR downstream signaling leads to constriction of peripheral airways, and the process involves the activation of SphKs and release of intracellular Ca^2+^ levels (16). The systemic administration of S1P increased the airway resistance and cholinergic activity in whole mouse lung model (17). There was a marked increase on RANTES, CCR3, and IL-17 level after S1P treatment. Interestingly, S1P-mediated effects were abrogated by JTE-013 (an S1PR2 antagonist) or Y-27632 (an inhibitor of Rho kinase) indicating the role of S1PR2 (18, 19). This was further confirmed by silencing of S1PR2 using siRNA approach, which also showed the inhibition of S1P-driven Rho kinase activity. An inhibitor of SphK; SKI-II (4-[4-(4-chloro-phenyl)-thiazol-2-ylamino]-phenol) was tested for its effect on bronchial smooth muscle hyperresponsiveness and airway inflammation. It was observed that the inhibitor augmented the hyperresponsiveness but had no effect on airway inflammation (20). An isoenzyme specific inhibitor of SphK1 (SK1-I) decreased airway hyperresponsiveness (AHR) and inflammation in a mast cell-dependent murine model of allergic asthma (21). SK1-I attenuated the antigen-dependent activation of both human and mouse mast cells and also reduced the circulating level of S1P in both lung and circulation. Further, NF-kB activation is a prerequisite for the pro-inflammatory cytokine production after FcεRI activation. It was observed that SK1-I blocked the activation of p65 and reduced the phosphorylation of p65 at Ser276 position and was substantiated by the observation that TNF-alpha and IL-6 levels reduced after inhibitor treatment in ovalbumin challenged animals (21).

FTY720, an approved drug for multiple sclerosis, binds to all S1PRs except S1PR2 (22). Treatment with FTY720 suppressed both Th1- and Th2-driven lung inflammation. Further, this agonist inhibited the ovalbumin-induced bronchial hyper reactivity to methacholine in mice and was associated with a decrease in the lymphocyte and eosinophil count in bronchiolar alveolar lavage (23). Idzko et al. also confirmed the observation and provided more mechanistic insight for the action of FTY720 (24). The systemic administration resulted in an increase in the circulating dendritic cells along with an absence of lymphopenia and T cell retention in the lymph nodes. Studies were performed using isolated trachea from both mouse and rat where it was incubated with phospho-FTY720. The treatment increased the hyperresponsiveness to 5-hydroxytryptamine, and this effect was completely abolished in trachea isolated from S1PR3^−/−^ mice indicating the role of S1PR3 in AHR (25). The involvement of a specific population of neurons, present in the vagal ganglia, in AHR was also analyzed. These ganglions express TRPV1 ion channel and intensify broncho constrictions very similar to that seen in asthma in sensitized lungs (26). Transcriptomic analysis of these cells revealed one of the abundant transcripts as S1PR3 (26) and further confirmatory studies indicated that S1P is one of the signals involved in the cross talk between immune cell activity and neurons in asthmatic responses.

Infiltration of B cells is another hallmark of AHR after OVA sensitization. The pretreatment of lungs with SphK inhibitors attenuates the B cell mobilization, thereby linking S1P signaling to B cell recruitment. S1P-dependent AHR response is associated with an immune suppressive environment as seen from increased number of Treg cells. Monoclonal antibody-based depletion of CD20+ B cells in mice was associated with S1P-dependent AHR and inflammation. The recruitment of the B cells is a mechanism to counteract the S1P-dependent inflammatory environment in lungs (27).

Of note, it was recently reported that ORM (yeast)-like protein isoform 3 (ORMDL3) gene is associated with asthma susceptibility, based on the genome-wide association studies (28). Interestingly, FTY720 reduced the ORMDL3 expression, AHR and associated inflammation, and mucus production and elevated the ceramide levels in house dust mite-induced lung inflammation model in mice, further confirming the potential beneficial role of FTY720 in the treatment of asthma (29). Moreover, mice with increased expression of ORMDL were shown to have reduced levels of different types of sphingolipids (30).

Disodium cromoglycate (DSCG), a mast cell stabilizer, is used in the therapy of asthma and is included in British Thoracic Society guidelines. It was shown that DSCG inhibited asthma-like features induced by S1P in mice as seen from the reduced recruitment of mast cells and B cells to lungs, decrease in AHR, and inflammation (31).

### Lung Cancer

Lung cancer is one of the leading causes of cancer-related death worldwide. The role of SPL was investigated in the context of chemoresistance and the overexpression of this enzyme in A549 cells makes it more sensitive to cisplatin, which was mediated through upregulation of p38 and JNK pathway (32). Beneficial effects of FTY720 in reducing tumor burden in a murine model of urethane-induced lung cancer were also reported (33). S1PR3 expression was elevated in lung adenocarcinoma cell lines when compared to normal human airway epithelial cells. It was also noticed that signaling through S1PR3 increased the expression of EGFR and silencing of S1PR3 abolished the S1P-dependent EGFR activation (34). In non-small cell lung cancer (NSCLC) cells, the ectopic expression of Spns2, an S1P transporter, resulted in increased apoptosis through modulation of GSK-3β and Stat3 pathways (35). A combination of ABC294640, an inhibitor of SphK2, and tumor necrosis factor-related apoptosis-inducing ligand (TRAIL) increased the apoptosis in NSCLC cell lines *in vitro* (36). Silencing of Sphk2 showed similar effects with TRAIL, as shown by ABC294640 (36). Studies using fibroblasts showed that S1P in nucleus, produced mainly by SphK2, interacted with hTERT. Silencing either SphK2 or S1P binding pockets leads to decreased stability of hTERT and loss of telomere integrity (37). Genetical or pharmacological inhibition of SphK2 decreased the growth of lung tumor in mice. This study demonstrated the important role of S1P in maintaining telomere stability (37). Glucosylceramide synthase, in glycolipid biosynthesis, was shown to be over expressed in lung cancer and is implicated in chemoresistance (38). Inhibition of this enzyme enhanced the anticancer potential of ABC294640 in lung cancer (39). This study advocates the possibility of a combination of SphK2 inhibitors and GCS inhibitors in lung cancer treatment.

Mesothelioma is a resistant form of cancer, which primarily develops in the lining of the lungs. Sphingosine inhibited the growth of mesothelioma cell lines and induced cell cycle arrest at the G0/G1 through the inhibition of PKC-δ (40). The elevated expression of SphK1 in malignant pleural mesothelioma tumor samples and cell lines has been reported. There is upregulation of histone acetyl transferases and a decrease in the expression of cell cycle-dependent kinase inhibitor genes (41). In a mouse model of this disease, the granulomatous inflammation (which was considered as a nearly mesothelioma like symptom) was greatly attenuated in SphK1^−/−^ mice as compared to SphK1^+/+^ mice (41) indicating the possibility of targeting SphK1 for the treatment of mesothelioma. However, this study does not exclude the role of SphK2 in mesothelioma, which requires further investigations.

### Pulmonary Hypertension (PH)

The role of sphingolipids in PH is also being identified. SphK1 and S1P were elevated in lungs of patients with PH as well as in animal models of hypoxia-mediated pulmonary hypertension (HPH). There is an increased proliferation of pulmonary artery smooth muscle cells (PASMCs), and associated pulmonary vascular remodeling is observed during PAH. Elevated levels of S1P has been detected in the plasma of PAH patients (42). The SphK1^−/−^ mice were protected against HPH as seen from reduced right ventricular systolic pressure and less severe pulmonary vascular remodeling (43). Interestingly, there was no protective effect in SphK2^−/−^ against HPH indicating the beneficial role of SphK 1 inhibition in the treatment of PH (43). S1P promoted the PASMCs proliferation (44, 45) through S1PR2, and this effect was nullified in SphK1^−/−^ mice. The involvement of S1PR2 was further confirmed by employing JTE-012, which prevented HPH and vascular remodeling (44). The treatment of rats suffering from the late stage of PAH with SphK1 attenuated the disease severity and reduced the levels of circulating S1P (45). The macrophages have a tendency to accumulate near lung arterioles and express high levels of leukotriene B_4_ (LTB_4_), which triggers cell death in pulmonary artery endothelial cells. This effect was mediated through inhibition of SphK1–eNOS signaling (44). The blocking of LTB4 production reversed fulminant PH through the restoration of the SphK1–eNOS pathway. Hypoxic pulmonary vasoconstriction (HPV) is also a contributing factor for PH. Recently, the role of cystic fibrosis transmembrane regulator (CFTR) is highlighted in HPV. It was observed that neutral sphingomyelinase and hypoxia-induced pulmonary vasoconstriction were inhibited by genetic or pharmacological silencing of SphK1 or through antagonism of S1PR 2 and 4 (45). These studies effectively pointed out the importance of S1P signaling in PH and can be the molecular target in the treatment of PH.

### Cystic Fibrosis (CF)

Cystic fibrosis is a multisystem genetic disorder, which mainly affects the lungs. The study by Xu et al. (46) showed that the functionally impaired lung dendritic cells contribute to the development of CF. The decreased level of S1P in the BALF results in a reduced recruitment of dendritic cells to the lungs and also affects the activation. The exogenous addition of S1P or FTY720 to the CF BALF could restore the expression of MHCII and CD40. This effect seems to be mediated through S1PR as the addition of JTE-013 and VPC20319 (an S1PR1/3 agonist) brought down the expression of the activation markers. The dysfunction of CFTR alters immune cell responses, and CFTR is involved in cellular uptake of S1P. The reduced expression of CFTR in CF will lead to a reduced uptake of S1P. Thus, S1P would be available to bring in an exacerbated cycle of inflammation and angiogenesis as seen in CF (47). S1P inhibits the CFTR activity through AMPK (48) and controls its own degradation through CFTR conductance modulation that depends on AMPK and S1PR2. The expression of a mutated form of CFTR (ΔF508) is observed in a vast majority of CF patients and leads to an increased sphingolipid synthesis, which indicated that CFTR functions as a feedback system in sphingolipid biosynthesis (49). The level of S1P was found to be reduced in a mutant form of CFTR mouse. However, treatment with SPL inhibitor (LX2931) could restore the S1P levels. Moreover, the CF phenotype of CFTR mutant mice was partially corrected by LX2931 further confirming the possible therapeutic targeting of S1P signaling in CF (50).

### Pulmonary Fibrosis (PF)

Pulmonary fibrosis thickens the alveoli leading to the development of scars and is associated with severe breathing problems. SPL is reported to be an endogenous inhibitor of pulmonary fibrosis (51). The expression levels of SphK1 and SphK2 were found to be high in idiopathic pulmonary fibrosis patients, and the level of expression correlated with the severity of the disease. The knockdown of SphK1 protected mice from lung fibrogenesis. The levels of S1P and dihydro S1P were high in mice challenged with bleomycin. Administration of SK-II reduced the levels of the sphingolipids and protected against the disease and the disease associated mortality (52). A similar effect was seen in radiation-induced pulmonary fibrosis, where treatment with myriocin reduced the S1P and dihydro S1P levels, thereby reducing the onset of the disease (53). The EMT that is associated with PF has an increased level of S1P associated with it. S1P-mediated EMT through interaction with S1PR2 and S1PR3 and activates p-Smad3, RhoA-GTP, and TGF-β (54).

### Acute Lung Injury (ALI) or Acute Respiratory Distress Syndrome (ARDS)

Acute lung injury/acute respiratory distress syndrome is an acute respiratory failure associated with substantial morbidity and mortality to patients (55). Loss of Forkhead protein (FOXF1) in endothelial cells leads to ALI and administration of S1P reduced the lung edema and promoted survival (56). Further, knockdown of FOXF1 leads to increased expression of S1PR1 and thereby maintain the endothelial barrier integrity (56). Genetic screening of ALI subjects found a striking link between a single nucleotide polymorphism in cortactin gene and ALI. Cortactin is involved in maintaining barrier integrity. Interestingly, this polymorphism in cortactin was responsible for diminishing the barrier protective effects of S1P in endothelial cells making them more susceptible to ALI (57). SphK1 also afford protection against radiation-induced lung injury (RILI). SphK1^−/−^ mice were highly susceptible to RILI and S1P receptor agonists like FTY720, p-FTY720, and SEW2871 attenuated RILI (58).

Glucocorticoids are currently in trials for ALI. Treatment with glucocorticoids leads to the enhanced synthesis of SphK1 and S1P production. Conversely, silencing SphK1 expression attenuated the effects of glucocorticoids in ALI. SphK1^−/−^ mice were more susceptible for LPS induced vascular leakage and were associated with delayed clearance of histamine and poor recovery from anaphylaxis, and these adverse events were reversed by administering exogenous S1P (59). The role of S1PR2 has been investigated in mast cell-dependent passive systemic anaphylaxis and found that pharmacological or genetic silencing of S1PR2 reduced the antigen-induced lung perivascular edema and anaphylactic responses. Mast cells increased the T cell recruitment through S1PR2 signaling coupled to STAT3 pathway. This, in turn, leads to production of chemokines in acute pulmonary allergic responses. Studies using antibody against S1P and S1PR2 agonists demonstrated that chemokine secretion and recruitment of T cells were reduced, further confirming the role of S1PR2 (60). Cui et al. reported another mechanism for S1PR2 where it inhibited the signaling through Akt, eNOS, and nitric oxide production and protected the animals from anaphylactic shock (61).

### Chronic Obstructive Pulmonary Disease (COPD)

Chronic obstructive pulmonary disease is an umbrella term referring to chronic pathological conditions affecting the respiratory system (62). Cordts et al. reported the mRNA expression profiling of S1PRs in the lungs of COPD patients showed significant decrease in S1PR5 and proposed that this receptor can be a novel target for pharmacotherapy (63). mRNA expression profiles of different SphKs, S1PRs, SPL, and phosphatase were analyzed in COPD patients and found significant upregulation of SphKs, S1PR2, and S1PR5 (63). The role of S1P signaling in efferocytosis was also reported. Cigarette smoke-inhibited efferocytosis was significantly reversed by either S1P or FTY720 (64). These observations further support the notion that S1P pathway can be a potential therapeutic target in COPD.

### Influenza

Influenza viruses are seasonal human pathogens causing pandemic morbidity and mortality. S1P-signaling components have been shown to interfere with virus infection and replication. SPL overexpressing cells were found to be resistant for viral amplification as compared to vector transfected cells as evident from reduced viral titer. The SPL overexpression leads to rapid activation of both STAT1 and ERK pathways (65). Teijaro et al. reported that during influenza infections, S1PR1 agonism was effective in suppressing pro-inflammatory cytokine and chemokine production as well as innate immune cell recruitment (66). S1PR1 located mainly in the pulmonary endothelial cells and agonists such as CYM-5442, RP-002, and AAL-R (67) were effective in protecting the mice from lethal infection with influenza virus. Using a Ferret model of human 2009 pandemic influenza virus infection, it was observed that the S1PR1 agonist RP-002 was effective in alleviating clinical symptoms and lung pathology independent of virus replication. Importantly, a combination of RP-002 and oseltamivir (an antiviral drug) was more effective in blunting immune response and suppressing viral replication (68). Thus, this study provided the advantages of combining antiviral drugs and S1PR1 modulators in the treatment of influenza infection. The various effects mediated by S1P system in different lung disorders are summarized in Table 1.

**Table 1:** **Sphingosine 1-phosphate (S1P) signaling in lung disorders: the effects of different inhibitors of SphKs, S1P receptor modulators, and S1P antibody in different diseases related to lung with possible mechanism of action**.

S. no.	Drug used	Disease	Model	Mechanism of action	Reference
1	JTE-013	Asthma	Human bronchial smooth muscle (BSM) cells	Suppression of S1P-induced inhibition of RANTES production	(18)
2	JTE-013	Asthma	Mouse model of allergic airway inflammation	Inhibition of S1P-mediated BSM contraction	(19)
3	SKI-II	Asthma	Ovalbumin-sensitized mouse model	Alleviation of BSM hyperresponsiveness	(20)
4	Inhibitor of SphK1	Asthma	Mast cell-dependent mouse model of ovalbumin-induced asthma	Inhibition of antigen-dependent mast cell activation and NF-κB activation	(21)
5	FTY720	Asthma	Antigen-sensitized murine asthma model	Inhibition of Th1- and Th2-mediated airway inflammation, inhibition of T cell, and eosinophil infiltration into bronchial tissue	(23)
6	FTY720, S1P	Asthma	Mouse asthma model	Suppression of Th2-dependent eosinophilic airway inflammation and bronchial hyperresponsiveness	(24)
7	SphKI/II inhibitor	Asthma	OVA-sensitized BALB/c mice	Inhibits phosphorylation of sphingosine and reduced B cell infiltration into lungs	(28)
8	FTY 720	Asthma	House dust mite model of allergic lung inflammation in C57BL/6J mice	Attenuates ORMDL3 expression	(29)
9	FTY 720	Lung cancer	Urethane-induced lung cancer in BALB/c mice	Decreased PCNA, increased caspase expression, and impaired tumor development	(33)
10	ABC294640	Lung cancer	Non-small cell lung cancer cell lines	Augmentation of antitumor effect of tumor necrosis factor-related apoptosis-inducing ligand and upregulation of death receptor expression	(36)
11	ABC294640	Lung cancer	A549 xenografts in SCID mice	Reduced hTERT expression	(37)
12	Sphingosine	Mesothelioma	Mesothelioma cell lines	Inhibition of PKC-δ and induction of cell cycle arrest	(40)
13	JTE-013	Pulmonary hypertension (PH)	Hypoxia-mediated PH model	Prevention of development of hypoxia-mediated pulmonary hypertension	(42)
14	SKI2	PH	Hypoxia-mediated PH model in rodents	Reduced right ventricular systolic pressure, right ventricular hypertrophy, and pulmonary remodeling	(42)
15	SKI-II	PH	Pulmonary arterial smooth muscle cells	Attenuation of hypoxia-induced increase in pulmonary arterial pressure	(45)
16	JTE-013	Hypoxic pulmonary vasoconstriction	Perfused murine lungs	Reduction in pulmonary arterial pressure	(45)
17	JTE-013	Cystic fibrosis (CF)	Lung dendritic cells	Reduced expression of MHCII and CD40	(46)
18	VPC23019	CF	Lung dendritic cells	Reduced expression of MHCII and CD40	(46)
19	LX2931	CF	Cystic fibrosis transmembrane regulator mutant mice	Increases level of S1P, normalization of the MoDC/cDC ratio, reduction in T and B cells, and normalization of pro-inflammatory cytokine levels	(50)
21	Myriocin	PF	Mouse model of radiation-induced pulmonary fibrosis	Blocks sphingolipid *de novo* biosynthesis and reduced level of SphK1 and serine palmitoyl transferase	(53)
22	S1P	Acute lung injury (ALI)	Conditional FOXF1 knock out mice model	Restored endothelial barrier function and decreased lung edema	(56)
23	SEW2781	ALI	LPS induced vascular leakage	Reduced vascular leakage and strengthening of endothelial barrier	(59)
24	Sphingomab	Anaphylaxis	Antigen-induced allergic response murine model	Inhibition of mast cell activation and reduction in histamines, cytokines, and chemokines	(60)
25	JTE-013	Anaphylaxis	Antigen-induced allergic response murine model	Inhibition of mast cell activation and reduction in histamines, cytokines, and chemokines	(60)
26	CYM-5442, RP-002, AAL-R	Influenza	C57BL/6 mice infected with influenza virus	Reduction in cytokine and chemokine production, inhibition of macrophage, and natural killer cell accumulation in lungs	(66)

## Conclusion

Sphingolipids are known to be involved in the development and progression of diseases. The level of SphK1 and S1P is elevated and often correlates with the disease severity. The abrogation of the enzyme and its product was found to lead to a reduction in the disease symptoms. The review thus summarizes the effects mediated by the SphK/S1P axis and stresses the importance of developing therapies targeting the signaling of sphingolipids, and hence the effective treatment of several pulmonary diseases.

## Author Contributions

All authors listed have made substantial and intellectual contribution in the preparation of the manuscript, and final version was approved for publication.

## Conflict of Interest Statement

The authors declare that the research was conducted in the absence of any commercial or financial relationships that could be construed as a potential conflict of interest.
