# Corrigendum: Sphingosine 1-Phosphate: A Novel Target for Lung Disorders

**DOI:** 10.3389/fimmu.2018.01628

**Published:** 2018-07-17

**Authors:** Sabira Mohammed, K. B. Harikumar

**Affiliations:** ^1^Cancer Research Program, Rajiv Gandhi Centre for Biotechnology, Thiruvananthapuram, India; ^2^Manipal Academy of Higher Education, Manipal, India

**Keywords:** FTY720, S1PR, asthma, lung diseases, sphingosine 1-phosphate, sphingosine kinase

In the published article, there was an error regarding the affiliation(s) for Sabira Mohammed. Dr Mohammed should also have an additional affiliation:

Manipal Academy of Higher Education, Manipal, India.

The authors apologize for this error and state that this does not change the scientific conclusions of the article in any way.

The original article has been updated.

## Conflict of Interest Statement

The authors declare that the research was conducted in the absence of any commercial or financial relationships that could be construed as a potential conflict of interest.

